# Bilateral adrenalectomy in the context of primary adrenal insufficiency due to colorectal cancer metastasis

**DOI:** 10.3332/ecancer.2022.1395

**Published:** 2022-05-23

**Authors:** Joaquin Fernandez Alberti, Walter S Nardi, Maricel Recalde, Sergio D Quildrian

**Affiliations:** 1 Department of General Surgery, Buenos Aires British Hospital, Perdriel 74, Buenos Aires C1280AEB, Argentina; 2 Retroperitoneal, Pelvic and Adrenal Unit, Department of General Surgery, Buenos Aires British Hospital, Perdriel 74, Buenos Aires C1280AEB, Argentina; 3 Department of Endocrinology, Buenos Aires British Hospital, Perdriel 74, Buenos Aires C1280AEB, Argentina

**Keywords:** adrenal insufficiency, bilateral metastasis, adrenalectomy, surgical oncology, colorectal cancer

## Abstract

**Introduction:**

Adrenal glands are a common site of metastasis for several types of malignancies. Nevertheless, bilateral metastasis leading to adrenal insufficiency is a very rare presentation.

**Presentation of case:**

We present a 62-year-old woman with previous history of colorectal cancer and bilateral adrenal metastasis associated with primary adrenal insufficiency. The patient underwent bilateral open adrenalectomy after a multidisciplinary tumour board evaluation.

**Conclusion:**

The incidence of adrenal insufficiency may be underestimated in patients with a history of cancer. Adrenal function must be evaluated in those patients presenting with bilateral adrenal masses and hormonal replacement therapy should be considered, if appropriate. In selected cases, bilateral adrenalectomy can give a possible therapeutic option for patients with confined disease to the adrenal glands.

## Introduction

The adrenal glands are commonly infiltrated with metastasis in patients with a variety of neoplastic diseases. The most frequent primary cancers that metastasise to the adrenals are lung (42%), breast (58%), melanoma (50%), gastric (16%), colorectal (14%) and oesophageal (10.3%) among others [1].

Although bilateral metastases are more common than unilateral, in most cases they do not lead to adrenal insufficiency. It develops when more than 90% of the adrenal tissue is destroyed, resulting in near to complete glucocorticoid deficiency often accompanied by a mineralocorticoid deficiency [2]. Moreover, it is rare for a patient to present with primary adrenal insufficiency secondary to bilateral adrenal gland metastases from a colorectal tumour; in fact, less than ten cases have been published in the English literature [3–8]. Failure to recognise chronic or acute adrenal insufficiency or epinephrine excess can cause significant morbidity or mortality [9].

## Case presentation

**Figure 1.**
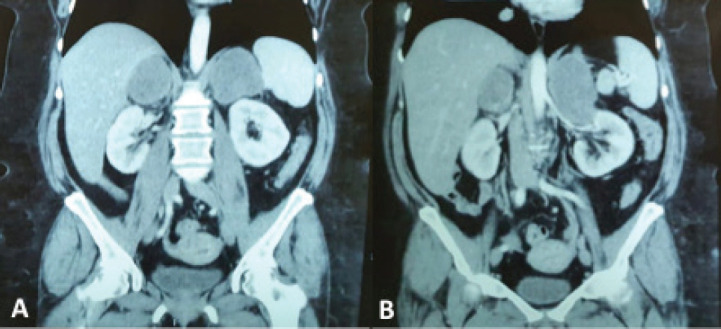
(a and b) Coronal sections on computed tomography (CT) scans showing bilateral adrenal masses (right of 66 mm and left of 106 mm).

A 62-year-old woman with a previous history of colorectal cancer was referred to our surgical department for presenting bilateral adrenal masses found on an abdominal CT scan at month 9 follow-up (Figure 1), associated with increasing levels of adrenocorticotropic hormone (ACTH) (last value: 320 pg/ml; normal value: <54 pg/ml) and low levels of dehydroepiandrosterone (86 ng/ml; normal value: 336–2,490 ng/ml). Her medical history revealed a laparoscopic right hemicolectomy for a colorectal cancer (pT4 N0 M0, pStage II) without requiring any adjuvant therapy.

**Figure 2.**
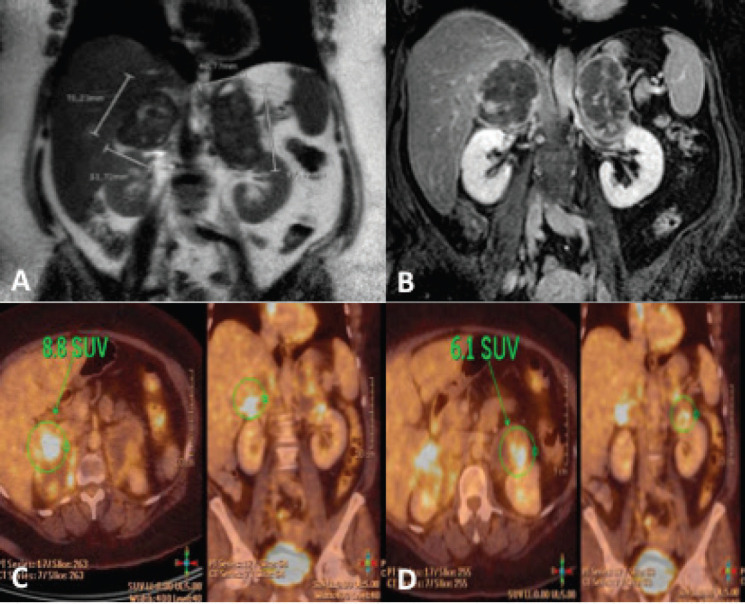
(a) Coronal section on MRI in T2-weighted sequence showing bilateral adrenal masses (right of 75 × 52 mm and left of 98 × 49 mm). (b) Similar section in T1-weighted with fat suppression and Gadolinium sequence. (c and d) PET-CT showing peripheral metabolic increase in nodular formations in right and left adrenal glands, with 8.8 and 6.1 SUV, respectively.

Physical examination evidenced morbid obesity (body mass index (BMI): 49 kg/m^2^) and skin hyperpigmentation. She also referred long-time tiredness and weight gain. Laboratory tests evidenced a primary adrenal insufficiency associated with elevated tumour markers (carcinoembryonic antigen (CEA): 40.9 ng/ml; normal value: 1–5.2 ng/ml). Oral corticosteroid replacement was initiated (hydrotisone 20 mg and fludrocortisone 0.05 mg daily) showing immediate symptom improvement. Also, a central nervous magnetic resonance imaging (MRI) was requested which showed no abnormalities in the pituitary gland and pheochromocytoma was ruled out in laboratory tests. Abdominal MRI is shown in Figure 2a and b. PET-CT was also performed showing hypermetabolic bilateral adrenal masses with no systemic disease (Figure 2c and d).

**Figure 3.**
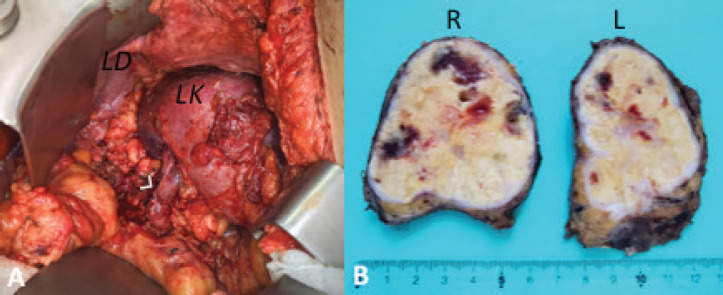
(a) Left surgical site post-adrenalectomy. Arrow: left renal vein; LK: left kidney; LD: left diaphragm. (b) Right (R) and left (L) adrenal glands sectioned.

After discussing the case in a multidisciplinary tumour (MDT) meeting, multiple CT-guided core biopsies were performed with a 14G needle using a posterior approach. Because of the impossibility to establish a definitive diagnosis on an imaging basis only, pathological assessment remains the gold standard of diagnosis. Differential diagnosis includes lymphoma, hyperplasia, hematoma, histoplasmosis and pheochromocytoma among others. As mentioned, a percutaneous CT-guided core needle biopsy was performed using the retroperitoneal approach. This route shows a minimal risk of seeding by preserving the posterior parietal peritoneum. Histopathology findings were primarily related to colorectal lineage, associated with patient’s cancer history. Based on this result, the decision was made for complete surgical resection. The patient underwent open bilateral adrenalectomy with two subcostal incisions (Figure 3). We chose to go with an open approach because of the size of both tumours and their relationship with renal vessels. She was discharged on post-operative day 6 without complications. Final histopathological examination confirmed a massive involvement by adenocarcinoma with multiple sites of necrosis in both surgical specimens.

In a new MDT meeting, it was decided to complete treatment with six sessions of chemotherapy (Capecitabine). At 1-year follow-up, she had normal CEA levels and abdominal CT scan and PET-CT without evidence of disease recurrence.

## Discussion

Colorectal cancer is one of the most frequent cancers worldwide. One of the first steps to initial staging includes abdominal/pelvis and chest computed tomography (CT) to evaluate for metastatic disease which occurs in approximately 20% of the patients at the time of diagnosis [10]. The most common sites of metastases are the lungs, liver and peritoneum [10]. Adrenal metastases can be seen in up to 14% of the patients with advanced colorectal cancer [3], indicating widespread disease; and, hence, patients with a solitary adrenal metastasis from colorectal carcinoma are rare, especially coupled with metachronous contralateral adrenal metastasis. Bilateral metastases are more commonly seen than unilateral ones and, in most cases, they do not lead to adrenal insufficiency. In order for this to happen more than 90% of the adrenal tissue needs to be destroyed. Adrenal insufficiency secondary to any type of metastatic cancer has been reported in fewer than 100 cases in the literature [11].

A metastatic origin should always be considered in a patient presenting with an adrenal mass and a history of malignancy. Evaluation of patients must include imaging with abdominal CT scan or MRI and be compared with a previous scan. In addition, a biochemical assessment to rule out a functional adrenal tumour needs to be performed. There are imaging features to be considered such as size >4 cm, growth in size >1 cm on serial imaging (within 1 year), irregular shape, heterogeneous and poorly defined margins, necrosis, Hounsfield unit >10 on non-contrasted imaging, <60% washout on CT imaging and hyperintense on MRI T2-weighted imaging [12].

Serum CEA is thought to be essential in predicting the presence of metastatic disease in the post-operative period of colorectal cancer [13]. In the case presented, imaging findings were associated with elevated serum CEA levels, making the scenario highly suspicious of bilateral metastasis. However, the multidisciplinary committee agreed to confirm the diagnosis with a percutaneous CT-guided biopsy. This practice is recommended for selected cases if the expected findings are likely to alter the management of the individual patient and after biochemical exclusion of catecholamine-producing tumours to help prevent potentially life-threatening complications [14].

Although prospective data are generally lacking, multiple retrospective investigations have demonstrated that adrenalectomy in highly selected patients with isolated or oligometastatic disease from primary sites, including the lungs, melanoma and kidney, can result in improved overall survival (OS) compared with similar patients who do not undergo adrenalectomy [12]. Several factors associated with poor survival outcome have been identified, such as the presence of active disease at the time of adrenalectomy, type of primary, disease-free interval, size of metastases and adrenalectomy bed recurrence [15–17]. In terms of OS, it is important to highlight the improvement observed in the past decades by the group of Memorial Sloan Kettering. They reported a 5-year OS of 24%, 29%, 31% and 40% in four consecutive series published between 1998 and 2018 [15, 18, 19]. These results probably represent better patient selection and improvements in systemic therapy.

Other alternatives to surgery have been described such as radiofrequency ablation mainly in patients who are poor surgical candidates. This treatment modality is used to ablate tumours (both benign and malignant) in a variety of tissues, including liver, lungs, bone, breast or kidney, and proved to be effective, safe and technically feasible. Mayo-Smith and Dupuy [20] presented the results from percutaneous ablation in 11 patients with metastatic lesions to the adrenal glands with a mean tumour size of 3.9 cm. Nonetheless, there are few reports of its application to adrenal tissue in the clinical scenario presented in our case report with bilateral and large tumours. No guidelines for adrenal ablation are established in the American or European literature.

The survival rate of patients with cancer is increasing, and improvements in imaging techniques have resulted in increased promptly recognition of adrenal gland metastases. However, the incidence of adrenal insufficiency may be underestimated, especially in patients with multiple metastases. In agreement with Crisci *et al* [7], we think that patients with multiple metastases from a known primary that complains of systemic symptoms must be evaluated for adrenal function and provided with a hormonal replacement therapy, if necessary.

## Conclusion

In conclusion, it seems reasonable to offer adrenalectomy to carefully selected patients with solid tumours and adrenal metastases, mainly patients with metachronous presentation (>6 months). The indication of LA must be made on a case-by-case evaluation in the context of a MDT board.

## Conflicts of interest

None declared.

## Funding

The authors have not declared a specific grant for this research from any funding agency in the public, commercial or not-for-profit sectors.

## Take home messages

- It is important to suspect potential adrenal malignancy in patients with oncological background. CT scanning and serum markers are useful tools in the postoperative period.
- Patients with multiple metastases from a known primary that complains of systemic symptoms must be evaluated for adrenal function and provided with a hormonal replacement therapy, if necessary. Adrenal insufficiency should not be underestimated.
- Laparoscopic adrenalectomy in these scenarios can be offered to selected patients after a MDT board discussion.

## Patient’s perspective

Oncologists, surgeons and endocrinologists meet with me every 3 months for my follow-up as a multidisciplinary team. I have direct communication with them in case of any need or doubt. I have felt very comfortable and secure during the treatment and follow-up so far.
